# Sport motivation is associated with lower aggression via emotional intelligence and self-control: a serial mediation study in undergraduates

**DOI:** 10.3389/fpsyg.2026.1762835

**Published:** 2026-02-26

**Authors:** Lili Wang, Jiankang Jia, Yucheng Jiang

**Affiliations:** School of Physical Education, Shihezi University, Shihezi, Xinjiang, China

**Keywords:** aggression, emotional intelligence, self-control, serial mediation, sport motivation

## Abstract

**Background:**

Whether sport engagement is associated with lower aggression remains contested. Less is known about how sport motivation, that is, reasons for engaging in sport, relates to aggression via emotion- and self-regulatory resources. These psychological pathways are rarely examined within a unified framework.

**Methods:**

In a cross-sectional survey, 485 students (18–25 years) from a public university completed validated Chinese versions of the Sport Motivation Scale II, the Wong and Law Emotional Intelligence Scale, the Self-Control Scale, and the Brief Aggression Questionnaire. Mediation was tested using Hayes’ PROCESS (Model 6; serial mediation with EI → SC) with percentile bootstrapping (5,000 resamples), controlling for sex and age.

**Results:**

Sport motivation was negatively associated with aggressive behavior (total effect: *B* = −0.267, 95% CI [−0.342, −0.193]; *β* = −0.303). When emotional intelligence and self-control were included, the direct association remained significant (*B* = −0.115, 95% CI [−0.188, −0.042]; *β* = −0.131). Indirect effects were significant via emotional intelligence (*B* = −0.068, 95% CI [−0.109, −0.030]), via self-control (*B* = −0.038, 95% CI [−0.070, −0.008]), and through emotional intelligence then self-control (serial indirect: *B* = −0.047, 95% CI [−0.071, −0.028]). Indirect pathways accounted for 56.87% of the total association. Sensitivity analyses using alternative operationalizations of sport motivation (SMS-II subscales and autonomous/controlled indices), EI branches, and self-control facets yielded consistent inferences for the serial indirect effect; the direction reversed for raw-scored amotivation.

**Conclusion:**

In this undergraduate sample, sport motivation was associated with lower aggression partly via a hypothesized serial indirect association through emotional intelligence and self-control (EI → SC), even after adjusting for sex and age and across alternative operationalizations of sport motivation. Given the cross-sectional self-report design, the findings should be interpreted as associational; longitudinal and experimental studies are needed to test temporal ordering and causal mechanisms.

## 1 Introduction

University students remain a population of concern for emotional and behavioral risks. The World Health Organization reports that roughly one in seven adolescents experiences a mental disorder, and suicide ranks among the leading causes of death in those aged 15–29; while not identical to aggression, these figures underscore a public-health need around emotion dysregulation, peer conflict, and risk-behavior management in educational settings (World Health Organization, 2025). In China, the General Office of the CPC Central Committee and the General Office of the State Council call for at least 1 h of daily physical activity and a strengthened system of physical-fitness monitoring, embedding sport within routine education (General Office of the CPC Central Committee, and General Office of the State Council, 2020). In parallel, the United Nations Educational, Scientific and Cultural Organization’s global report on school violence and bullying advocates whole-school, systemic approaches to violence prevention and student well-being, treating structured, routine school activities as key levers (UNESCO, 2019). Together, these policy signals indicate both the necessity and feasibility of linking campus sport with psychological and behavioral safety agendas in higher education.

Whether and how sport engagement is associated with aggression, however, remains contested. Meta-analytic evidence synthesizing randomized trials suggests that sport-based interventions are generally associated with reduced aggression, with stronger and more consistent effects for non-contact, cooperative, and well-supervised programs (Yang et al., 2023). At the same time, some longitudinal studies indicate that participation in highly confrontational sports such as rugby or boxing may be followed by greater off-field involvement in real-world conflicts (Kreager, 2007; Endresen and Olweus, 2005). This heterogeneity appears to reflect differences in sport type, degree of organization and oversight, measurement approaches, sample characteristics, and the surrounding climate (for example, the salience of competition and winning). Moreover, much of the evidence comes from children and adolescents, leaving questions about applicability to university settings. Conceptually, prior explanations often center on a single psychological pathway—such as emotion regulation or impulsivity—yielding fragmented accounts and diffuse intervention targets. Recent reviews and meta-analyses therefore call for clarifying the key links from sport to psychological resources to aggression in order to guide precise course and classroom design (Newman et al., 2021; Ouyang and Liu, 2023; Yang et al., 2023).

Against this backdrop, a mechanism-oriented perspective shifts attention from sport engagement per se to individuals’ reasons for engaging in sport/physical activity—namely, sport motivation—and evaluates whether the observed associations are consistent with an integrated motivation–emotion–control framework. Drawing on Self-Determination Theory, the process model of emotion regulation, and self-control perspectives embedded in the General Aggression Model, the present study tests a theory-driven serial mediation model in which sport motivation is associated with aggressive behavior indirectly via emotional intelligence and self-control. Because our data are cross-sectional, the proposed ordering (EI → SC) should be interpreted as a theory-driven, model-implied sequence rather than evidence of temporal precedence. Alternative directions remain plausible, including reverse or selection effects (e.g., lower aggressive tendencies may be associated with more sustained sport engagement and may be reflected in higher sport motivation) and reciprocal coupling between emotion-related capacities and inhibitory control (e.g., higher self-control may facilitate emotion regulation and thus be reflected in higher self-reported emotional intelligence). For parsimony and to align with our hypotheses focused on global SM, EI, SC, and AB, the primary analyses used observed mean composite scores. Sensitivity analyses were conducted and are reported in Supplementary Tables S2–S4.

### 1.1 Sport motivation and aggressive behavior

Self-Determination Theory (SDT) proposes that empowering, task-involving climates support the internalization of sport motivation, whereas controlling, performance-pressuring climates are more likely to be associated with controlled forms of motivation. In line with this view, studies in sport settings have reported opposite links between climate and aggressive conduct: among collegiate athletes, disempowering climates are associated with higher aggression while empowering climates are associated with lower aggression, with reduced moral disengagement emerging as a mediator (Zhong and Wang, 2025; Gürpınar, 2023); at the team level, coaches’ autonomy support has been linked to stronger collective efficacy which, in turn, is associated with lower aggression, and the association between autonomy support and aggression is attenuated once efficacy is included (Kim and Choi, 2024). Because motivational climate is theorized to relate to the quality of sport motivation (autonomous/internalized vs. controlled) and to broader norm adherence and emotion–behavior tendencies, these findings provide contextual support for an individual-level association between sport motivation and aggression. At the same time, the association is contingent on sport type and population characteristics: converging surveys indicate that men, full-contact sports, and higher competitive tiers show elevated physical aggression and antisocial behavior (Mazzanti et al., 2025; Bovolon et al., 2024; Vertommen et al., 2022), and national data suggest that a substantial proportion of adolescents report exposure to psychological or physical violence within competitive systems (Parent and Vaillancourt-Morel, 2021); within-team dynamics also matter, as stronger positional competition and a violent motivational climate have been prospectively associated with later aggression (Worley et al., 2022; González-Hernández et al., 2025). Mechanistically, meta-analytic evidence shows a moderate-to-strong association between moral disengagement and antisocial behavior (Zhu et al., 2025), and longitudinal studies suggest that higher baseline self-compassion is associated with faster declines in antisocial behavior across a season and with a weaker positive association between narcissism and aggression (McEwan and Zhang, 2024; Zhang et al., 2024). Notwithstanding, much of the literature treats contextual/climate routes or moral/emotional pathways in isolation; few investigations simultaneously consider emotion and control channels within the same model before re-examining the direct sport-motivation–aggression link (i.e., reasons/quality of engagement rather than participation volume or sport type), and evidence in general undergraduate samples remains limited. Accordingly, we hypothesize H1: sport motivation is negatively associated with aggressive behavior.

### 1.2 Emotional intelligence as a mediator

The Process Model of Emotion Regulation partitions regulation into situation selection, attentional deployment, cognitive reappraisal, and response modulation; the more proficient individuals are with antecedent-focused strategies—especially reappraisal—the better they may be able to dampen negative arousal before impulses form (Gross, 2015). Building on this, the ability model of emotional intelligence (EI) specifies four branch skills—perceiving, using, understanding, and regulating emotion—proposing that higher EI is associated with greater use of reappraisal relative to late-stage suppression, which may relate to fewer aggression-relevant responses (Mayer et al., 2016). From a motivational standpoint, Self-Determination Theory (SDT) suggests that autonomous motivation is linked to more positive affect and stable engagement opportunities, which may support the acquisition and enactment of emotional skills; within the General Aggression Model (GAM), such skills may reduce aggressive responding by shaping appraisal and response selection processes (Laborde et al., 2016).

Empirical work aligns with these linkages on both segments of the chain connecting sport and aggression through EI. In university and secondary-school samples, either higher physical-activity frequency or stronger intrinsic motivation correlates positively with EI facets such as clarity and repair (Wang et al., 2020; Vaquero-Solís et al., 2020), and a cross-cultural survey indicates that athletes exhibit higher EI and that EI covaries positively with autonomous motivation (Rogaleva et al., 2024). At the curricular level, reviews of cooperative or reflective physical-education programs consistently report gains in EI accompanied by reductions in misconduct or aggression (Rico-González, 2023). On the EI-to-aggression link, undergraduates with higher EI score lower across multiple violence dimensions (Lindell-Postigo et al., 2023); mechanistic studies further suggest that EI is indirectly associated with lower aggression via higher positive affect, lower negative affect, and stronger emotion-regulation self-efficacy (Chamizo-Nieto et al., 2020; Mesurado et al., 2018; Gao et al., 2023). Classroom-based EI training has been reported to yield sustained decreases in verbal and physical aggression at follow-up (Castillo-Gualda et al., 2018), and in competitive contexts, EI attenuates the positive association between narcissism and aggression (Jang et al., 2025). Notably, most studies test only one segment—either sport/motivation to EI or EI to aggression—rather than estimating mediation within a single model that simultaneously includes sport motivation, EI, and aggression; moreover, differences between ability-based and trait EI measures and questions of directionality remain to be adjudicated in unified analyses. The present study tests a theory-driven indirect pathway, while acknowledging that temporal ordering cannot be established with cross-sectional data. Accordingly, we hypothesize H2: emotional intelligence accounts for an indirect association between sport motivation and aggressive behavior in the hypothesized model.

### 1.3 Self-control as a mediator

The strength model of self-control conceptualizes self-control (SC) as a trainable yet depletable “mental muscle,” suggesting that emotionally driven impulses may be more likely when regulatory resources are low (Englert, 2016). Within the revised General Aggression Model (GAM), SC is posited to function as a brake at the appraisal–decision stage; once affect is activated, insufficient inhibition may increase the likelihood of aggressive output (Allen et al., 2018). A social-learning–self-discipline perspective adds that sport contexts may provide opportunities to practice SC through rule reinforcement, delay of gratification, and immediate feedback, while acknowledging that in climates emphasizing retaliation or dominance, these capacities may be co-opted in service of instrumental or premeditated aggression (Chester, 2024). Framed around impulsive aggression, higher-quality sport motivation—more autonomous, internalized, and stable—may be linked to sustained participation and goal management, which may help build and maintain SC resources that, in turn, could interrupt the emotion-to-action transition toward aggression.

Evidence across levels of analysis is broadly consistent with this account. Longitudinal and chain models have reported that greater physical-activity engagement is prospectively associated with higher baseline self-control and slower growth in aggression (Park et al., 2017; Rohlf et al., 2018); in university and combat-sport samples, a “sport motivation/activity → self-efficacy → SC → aggression” pathway has been supported, with indirect effects accounting for nearly half of the total association (Yu et al., 2024; Xu A. et al., 2025; Xu T. et al., 2025; Chen et al., 2019). Additional evidence comes from interventions and meta-analytic work: a meta-analysis of martial-arts programs indicates small-to-moderate reductions in aggression among children and youth (Harwood-Gross et al., 2017), while randomized martial-arts training, multicomponent programs, and sports-based courses for juvenile offenders have been shown to improve self-control and reduce both proactive and reactive aggression (Fung and Lee, 2018; Lok et al., 2024). Two meta-analyses and two systematic reviews converge that programs embedding SC exercises and rule reinforcement yield the largest effects, with average impacts in the range of −0.25 to −0.41 on Hedges’ *g* or Cohen’s *d*; non-contact formats tend to perform best (Spruit et al., 2016; Castillo-Eito et al., 2020; van der Sluys et al., 2024; Yang et al., 2023). At the same time, context matters: generic smartphone apps or brief drills show inconsistent benefits for aggression in general populations (Kip et al., 2021; Beames et al., 2023). Moderator findings are accumulating—years or rank in martial arts relate to higher SC and lower aggression (de Lorenco-Lima et al., 2025; Zvi and Lavi, 2025); in rural youth, exercise links to lower aggression via a “psychological capital → SC” sequence (Xu A. et al., 2025; Xu T. et al., 2025b); and in correctional settings, the inclusion and intensity of self-control and emotion-management modules are positively associated with the effectiveness of sports programmes in reducing reoffending (Jugl et al., 2023). Notably, much of this literature tests only one segment—either “sport → SC” or “SC → aggression”—often relies on self-report, and focuses on martial-arts or child samples; studies that simultaneously include sport motivation, SC, and aggression with comparable standardized effects in general undergraduate populations remain scarce. The present study tests a theory-driven indirect pathway involving self-control, while acknowledging that temporal ordering cannot be established with cross-sectional data and that self-control may also precede sport motivation. Accordingly, we hypothesize H3: self-control accounts for an indirect association between sport motivation and aggressive behavior in the hypothesized model.

### 1.4 Serial mediation of emotional intelligence and self-control

The process model of emotion regulation proposes that individuals with higher emotional intelligence (EI) may be better able to “cool down” earlier in the regulatory sequence—through situation selection, attentional deployment, and cognitive reappraisal—potentially reducing inhibitory load during response execution (Gross, 2015). The strength model of self-control (SC) emphasizes that control resources are limited yet trainable; effects of SC practice alone are modest but may be more reliable when coupled with emotion-regulation strategies (Friese et al., 2017). Within an SDT × GAM integration, higher-quality sport motivation may co-occur with positive affect and meaning while supplying rule-and-feedback cues, creating a context in which emotion–control coupling may operate (Boat and Cooper, 2019). Converging evidence from intervention and longitudinal designs is consistent with links between EI-related skills and inhibitory control: preschool programs combining EI with inhibition training have been reported to improve emotion recognition and impulse control and to reduce aggression (Romero-López et al., 2021); among collegiate athletes, EI training has been associated with fewer Go/No-Go commission errors alongside changes in prefrontal–parietal hemodynamics (Köroğlu et al., 2025). A three-wave cross-lagged panel study reported that emotional intelligence prospectively predicted lower levels of later problematic mobile social media use, with no evidence for the reverse path (Peng et al., 2025). A systematic meta-analytic review has reported that emotional intelligence is negatively related to health-related risk behaviors (Sánchez-López et al., 2022). In educational settings, PE programs that enhance students’ emotional intelligence have been reported to reduce verbal and emotional aggression (Ortiz-Franco et al., 2024), and similar protective patterns of higher EI with lower aggression are observed in athlete cohorts (Acebes-Sánchez et al., 2021). Taken together, these findings are broadly consistent with an ordering in which EI-related emotion-processing skills may support self-control processes and, in turn, relate to lower aggressive responses across populations and tasks; however, reciprocal relations between EI and SC are also plausible.

Evidence from a systematic review and meta-analysis of 17 EI-training interventions among healthcare workers suggests post-intervention improvements in emotional intelligence, although effect sizes may be inflated by methodological limitations (Powell et al., 2024); In clinical and elite-sport samples, higher emotional intelligence and related self-regulatory resources tend to co-occur with less dysregulated or risky behavior (Biolcati et al., 2025; Stanković et al., 2022). Recent work shows that higher emotional intelligence is associated with lower impulsivity and sensitivity to reward, which in turn are associated with less health-risk taking (Megías-Robles et al., 2023). From a resource-protection angle, higher EI is associated with better self-regulation, which in turn relates to fewer problematic behaviors such as smartphone addiction and poorer wellbeing (Mascia et al., 2020). Among lower-grade college students, exercise has been reported to relate to lower aggression through a “self-control → resilience” sequence (Yue et al., 2024). Over the life course, childhood SC forecasts broad midlife advantages in health and social functioning (Richmond-Rakerd et al., 2021), while community violence studies identify emotion-regulation breakdowns as an important correlate of aggression (Heleniak et al., 2018). Although the EI–SC–aggression sequence has been replicated across diverse settings, fewer studies have tested an explicit serial mediation while simultaneously including sport motivation, EI, SC, and aggression within a single model. Because the present data are cross-sectional, the EI → SC ordering is treated as a theory-driven, model-implied specification rather than evidence of temporal precedence. Accordingly, we hypothesize H4: emotional intelligence and self-control account for a serial indirect association between sport motivation and aggressive behavior in the hypothesized model, such that higher sport motivation is associated with higher EI, which is associated with higher SC, which is associated with lower aggression.

## 2 Methods

### 2.1 Participants

The target population comprised full-time undergraduates at Shihezi University who were not majoring in sport or physical education. Using randomized classroom intercepts stratified by academic building and time slot, the field team visited classrooms during routine teaching hours from November 1 to 5, 2025. Immediately after class, students present were invited to scan a QR code and complete an online questionnaire. The survey platform time-stamped each submission and generated an anonymous ID; average completion time was approximately 6–10 min. In total, 550 questionnaires were returned; based on two *a priori* exclusion criteria—completion time under 6 min and substantial item nonresponse—65 cases were removed, yielding a valid analytic sample of N = 485 and an overall validity rate of 88.2%. The sample included 231 men (47.6%) and 254 women (52.4%), ages 18–25 years.

The study protocol and informed consent form were reviewed and approved by the Science and Technology Ethics Committee of the First Affiliated Hospital of Shihezi University (Ethics Review No.: KJ2025-520-01) and complied with the Declaration of Helsinki. All participants provided electronic informed consent before beginning the survey. Data were collected anonymously without personally identifiable information and were used solely for academic research and instructional improvement; no data were disclosed to third parties without authorization.

### 2.2 Measurement

#### 2.2.1 Sport motivation scale

We assessed sport motivation (SM) using the validated Chinese version of the Sport Motivation Scale II (SMS-II; Pelletier et al., 2013; CSMS-II; Li et al., 2018). The instrument contains 18 items across six subscales—intrinsic motivation, integrated regulation, identified regulation, introjected regulation, external regulation, and amotivation—each with three items. The original response format is a 6-point Likert-type scale. To align with the other instruments and reduce respondent burden, we used a 5-point format (1 = “strongly disagree” to 5 = “strongly agree”). Prior work suggests that modest category compression can yield comparable reliability in many contexts, but altering response formats may affect score distributions, variances, effect-size estimates, and comparability with prior studies; therefore, related inferences should be interpreted cautiously (Preston and Colman, 2000). Prior work supports acceptable reliability and structural validity of the SMS-II in both English and Chinese samples. In the present sample (*N* = 485), internal consistencies for the six subscales were *α* = 0.86–0.88, and CFA indicated good fit (χ^2^/df = 1.880, RMSEA = 0.043, CFI = 0.987, TLI = 0.985), suggesting that the 5-point format preserved expected measurement properties. For the primary analyses, we reverse-scored the amotivation items and computed an overall SMS-II index as the mean across all 18 items, such that higher scores indicate higher overall motivation and lower amotivation. Because the SMS-II captures qualitatively distinct regulations, this 18-item mean was treated as a pragmatic overall motivation index rather than a pure autonomy score; autonomy-specific inferences were therefore primarily based on sensitivity analyses using each SMS-II subscale and the autonomous/controlled composites (Autonomous composite = mean of intrinsic motivation, integrated regulation, and identified regulation; Controlled composite = mean of introjected regulation and external regulation). In robustness checks, amotivation was examined in its raw direction as a separate predictor. The SMS-II contains no reverse-worded items; in this study, “reverse-scoring” refers only to recoding the amotivation items for the purpose of computing the overall 18-item mean score.

#### 2.2.2 Emotional intelligence scale

We assessed emotional intelligence (EI) with the Wong and Law Emotional Intelligence Scale (WLEIS; Wong and Law, 2002) and its Chinese adaptation (Shi and Wang, 2007). The instrument comprises 16 items covering four branches—Self-Emotion Appraisal (SEA), Others’ Emotion Appraisal (OEA), Use of Emotion (UOE), and Regulation of Emotion (ROE). Although the original uses a 7-point Likert-type format, we used a 5-point response scale (1 = “strongly disagree” to 5 = “strongly agree”) to harmonize response formats across instruments and reduce respondent burden. In pilot testing and in the present sample, internal consistency and CFA fit indices remained acceptable, suggesting that the category compression did not materially compromise measurement quality, although comparability with studies using the original response range should be interpreted with caution. Prior evidence in a Hong Kong undergraduate sample (*N* = 418) reported subscale Cronbach’s *α* values of 0.83–0.90 and acceptable CFA fit (χ^2^/df = 2.13, RMSEA = 0.053, CFI = 0.92, TLI = 0.91), while a three-university mainland sample (*N* = 1,458) yielded α = 0.79–0.88 (overall *α* = 0.86) with RMSEA = 0.045 and CFI = 0.97, supporting structural validity. An example item is “I have a good sense of why I feel certain feelings most of the time” (SEA). In the present sample (*N* = 485), the four subscales demonstrated *α* = 0.83–0.86, and CFA indicated good fit (χ^2^/df = 1.951, RMSEA = 0.044, CFI = 0.981, TLI = 0.978), suggesting that the 5-point format preserved expected psychometric properties. We computed an overall EI composite as the mean of the 16 items (higher scores reflect stronger emotion perception, utilization, and regulation) for subsequent correlation and mediation analyses.

#### 2.2.3 Self-control scale

We measured self-control with the Chinese 19-item college revision of the Self-Control Scale (SCS-C19; Tan and Guo, 2008), adapted from Tangney et al.’s 36-item SCS (Tangney et al., 2004). The SCS-C19 uses a 5-point Likert-type format from 1 (“strongly disagree”) to 5 (“strongly agree”). Items 1, 5, 11, and 14 are positively keyed, whereas all other items are reverse-keyed and were reverse-scored prior to computing composite scores. The instrument includes five facets—impulse control, healthy habits, resisting temptation, focus on work, and moderation in recreation—each represented by 3–6 items. Prior work with Chinese undergraduates (*N* ≈ 800) reported overall Cronbach’s *α* = 0.86 and acceptable CFA fit (χ^2^/df = 3.03, RMSEA = 0.050, CFI = 0.93), supporting reliability and factorial validity. An example item is “People say I am impulsive” (impulse control). In the present sample (*N* = 485), subscale alphas ranged from 0.76 to 0.89, and CFA indicated good fit (χ^2^/df = 2.023, RMSEA = 0.046, CFI = 0.977, TLI = 0.974), suggesting stable structure and internal consistency. After reverse-scoring the reverse-keyed items, we computed an overall SC composite as the mean of the 19 items (higher scores indicate stronger self-control) for use in the correlation and mediation analyses.

#### 2.2.4 Aggressive behavior scale

We assessed aggressive behavior (AB) using the bilingual Chinese–English Brief Aggression Questionnaire (BAQ; Webster et al., 2014). The BAQ contains 12 items tapping four facets—physical aggression, verbal aggression, anger, and hostility—with three items per facet. The original response format is a 7-point Likert scale from 1 (“extremely untrue of me”) to 7 (“extremely true of me”). To harmonize response formats across instruments and reduce respondent burden, we used a 5-point response scale (1 = “strongly disagree” to 5 = “strongly agree”), adapting the response anchors accordingly. Pilot testing and the present data indicated acceptable internal consistency and CFA fit; however, comparisons with studies using the original 7-point response range should be made with caution. Item 4 was reverse-keyed and was reverse-scored prior to computing composite scores. Higher scores reflect stronger aggressive tendencies. Prior work with U.S. undergraduates reported overall Cronbach’s *α* = 0.86, subscale *α* = 0.71–0.81, and acceptable CFA fit (χ^2^/df = 2.00, RMSEA = 0.060, CFI = 0.95, TLI = 0.94). An example item is “I will resort to force to defend my rights when necessary” (physical aggression). In our sample (*N* = 485), subscale alphas ranged from 0.85 to 0.87, and CFA showed good fit (χ^2^/df = 2.420, RMSEA = 0.054, CFI = 0.986, TLI = 0.982), supporting the expected four-factor structure with the 5-point format. After reverse-scoring Item 4, we computed an overall AB composite as the mean of the 12 items for the correlation and mediation analyses, with higher values indicating greater aggression.

Composite scoring rationale. Although the SMS-II, WLEIS, SCS-C19, and BAQ are multidimensional instruments, our primary hypotheses and mediation model focus on the overall (global) level of sport motivation, emotional intelligence, self-control, and aggressive tendencies rather than differential effects of specific subcomponents. Using observed mean composites (i) preserves interpretability of the serial mediation model, (ii) reduces model complexity and multiplicity of tests that would arise from simultaneously entering multiple correlated subscales/facets, and (iii) is consistent with our use of regression-based mediation in PROCESS. To address concerns that aggregation could obscure theoretically meaningful heterogeneity—especially for sport motivation and for EI/SC subdimensions—we conducted sensitivity analyses using theoretically informed alternative operationalizations. Results are reported in Supplementary Tables S2–S4.

### 2.3 Statistical analysis

**Figure 1:**
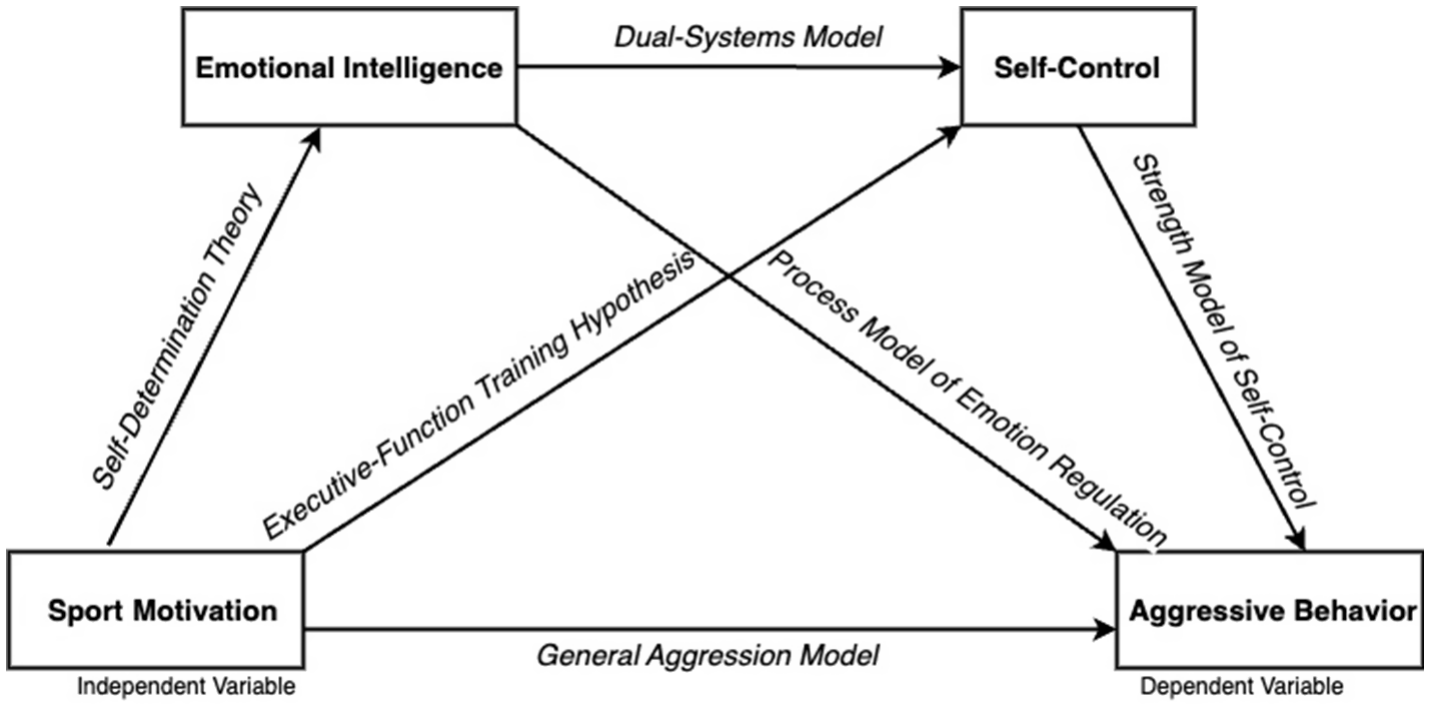
Conceptual framework and hypothesized serial mediation model linking sport motivation with aggressive behavior via emotional intelligence and self-control.

All analyses were conducted in SPSS 27.0 and AMOS 26; AMOS was used exclusively for confirmatory factor analyses (CFA) and evaluation of the measurement model fit. Following the scoring procedures described above (including reverse-scoring where applicable), we computed composite scores as mean item ratings for SM, EI, SC, and AB. Internal consistency was estimated with Cronbach’s alpha. Descriptive statistics included means, standard deviations, skewness, and kurtosis; bivariate associations were examined using two-tailed Pearson correlations with *α* = 0.05. Sex differences were tested with independent-samples t tests and accompanied by Cohen’s d. Age differences across four groups were evaluated with one-way ANOVA, reporting the F statistic and partial η^2^, followed by Tukey’s *post hoc* comparisons. Potential common-method variance (CMV) was assessed using Harman’s single-factor test based on an unrotated principal components solution and by comparing the fit of a one-factor CFA model with the hypothesized multi-factor measurement model. Mediation was tested in PROCESS v4.2 using Model 6 (serial mediation; EI → SC) with percentile bootstrapping (5,000 resamples). Model 6 provides estimates of the total and direct effects as well as the specific indirect effects via EI, via SC, and via the serial EI → SC pathway, which were used to evaluate H2–H4. Sex and age were included as covariates in all mediation models. We report unstandardized coefficients (B) with 95% confidence intervals for the total and direct effects, and 95% percentile bootstrap confidence intervals for specific indirect effects; indirect effects were considered statistically significant when the 95% percentile bootstrap confidence interval did not include zero. Standardized coefficients (*β*) are reported for the regression equations. Sensitivity analyses (alternative operationalizations). Sensitivity analyses were conducted to examine whether inferences were sensitive to the multidimensionality of the key constructs (Figure 1). Specifically, we re-estimated the serial mediation model (PROCESS Model 6; EI → SC; 5,000 bootstrap resamples; covariates = sex and age) using (a) each SMS-II subscale as X as well as autonomous and controlled motivation indices (and amotivation additionally examined in its raw direction), (b) each EI branch (SEA, OEA, UOE, ROE) as M1 while retaining overall self-control as M2, and (c) each self-control facet (IC, HH, RT, WF, AE) as M2 while retaining overall EI as M1. Results are summarized in Supplementary Tables S2–S4.

## 3 Results

### 3.1 Assessment of common-method variance

All measures were collected in a single session from the same respondents, which may raise concerns about common-method variance (CMV). Procedurally, the survey was anonymous and emphasized voluntary participation and that there were no right or wrong answers, which may reduce evaluation apprehension. Statistically, we conducted Harman’s single-factor test using an unrotated principal components analysis. Four factors had eigenvalues greater than 1, and the largest factor accounted for 36.76% of the total variance, below the commonly cited 40% benchmark. We also compared a one-factor confirmatory factor analysis (CFA) model (all indicators loading on a single latent factor) with the hypothesized multi-factor measurement model; the one-factor model fit substantially worse than the multi-factor model. Together, these checks suggest that a single common factor is unlikely to fully explain the observed pattern of associations; however, residual method variance cannot be ruled out in a single-source, self-report, cross-sectional design and is therefore considered in the limitations. Fit indices for the one-factor versus multi-factor CFA comparison are provided in Supplementary Table S1. Harman’s single-factor test is an insensitive diagnostic and does not rule out residual method variance.

### 3.2 Descriptive statistics and correlation analysis

Table 1 summarizes the sample (*N* = 485): Sport Motivation (SM) had *M* = 3.45, SD = 0.91; Emotional Intelligence (EI) M = 3.76, SD = 0.85; Self-Control (SC) *M* = 3.72, SD = 0.77; and Aggressive Behavior (AB) M = 2.14, SD = 0.80. Skewness ranged from −0.714 to 0.580 and kurtosis from −0.549 to 0.135 (absolute values < 1), indicating approximate normality and suitability for parametric analyses. Two-tailed Pearson correlations (*α* = 0.05) showed that SM correlated positively with EI (*r* = 0.451, *p* < 0.001) and SC (*r* = 0.313, *p* < 0.001), and negatively with AB (*r* = −0.349, *p* < 0.001). EI and SC were positively related (*r* = 0.462, *p* < 0.001), and both were inversely related to AB (EI–AB: *r* = −0.440; SC–AB: *r* = −0.518; both *p* < 0.001), with the SC–AB link largest in absolute magnitude. The maximum absolute correlation was |*r*| = 0.518, suggesting no collinearity concerns for subsequent regression-based models. Overall, higher SM was associated with higher EI and SC and with lower AB; the negative SM–AB association is consistent with H1 and provides descriptive context for the subsequent regression-based mediation analyses.

**Table 1:** Means, standard deviations, and correlations among study variables (*N* = 485).

Variable	*M*	SD	1	2	3	4
Sport motivation (SM)	3.45	0.91	1			
Emotional intelligence (EI)	3.76	0.85	0.451	1		
Self-control (SC)	3.72	0.77	0.313	0.462	1	
Aggressive behavior (AB)	2.14	0.80	−0.349	−0.440	−0.518	1

*N* = 485. Two-tailed tests. All correlations *p* < 0.001.

### 3.3 Demographic characteristics of the sample

Table 2 presents independent-samples tests by gender. Female students scored higher than male students on Sport Motivation (SM), Emotional Intelligence (EI), and Self-Control (SC), whereas males scored higher on Aggressive Behavior (AB). Specifically: SM (men 3.33 ± 0.91; women 3.57 ± 0.90), *t*(483) = −2.924, *p* = 0.004, Cohen’s *d* = −0.266 [−0.445, −0.087]; EI (men 3.55 ± 0.89; women 3.95 ± 0.76), variance homogeneity was violated and Welch’s correction was applied, *t*(453) = −5.288, *p* < 0.001, *d* = −0.485 [−0.665, −0.304]; SC (men 3.50 ± 0.75; women 3.92 ± 0.73), *t*(483) = −6.154, *p* < 0.001, *d* = −0.560 [−0.741, −0.378]; AB (men 2.31 ± 0.81; women 1.99 ± 0.76), *t*(483) = 4.569, *p* < 0.001, *d* = 0.415 [0.235, 0.595]. In this sample, women reported higher SM, EI, and SC and lower AB than men, indicating that sex was associated with key study variables; therefore, sex was included as a covariate in the mediation analyses.

**Table 2:** Gender differences in sport motivation, emotional intelligence, self-control, and aggressive behavior (*N* = 485).

Variable	Male (*n* = 231) M ± SD	Female (*n* = 254) M ± SD	*t*(df)	*p*	Cohen’s *d* [95% CI]
SM	3.33 ± 0.91	3.57 ± 0.90	−2.924 (483)	0.004	−0.266 [−0.445, −0.087]
EI	3.55 ± 0.89	3.95 ± 0.76	−5.288 (453)	<0.001	−0.485 [−0.665, −0.304]
SC	3.50 ± 0.75	3.92 ± 0.73	−6.154 (483)	<0.001	−0.560 [−0.741, −0.378]
AB	2.31 ± 0.81	1.99 ± 0.76	4.569 (483)	<0.001	0.415 [0.235, 0.595]

Two-tailed independent-samples *t*-tests; Welch’s df used for EI due to unequal variances. Cohen’s *d* = (male − female)/pooled SD; positive values indicate higher male scores.

Table 3 reports one-way ANOVAs across age groups (18 years *n* = 121; 19 years *n* = 109; 20 years *n* = 111; 21–25 years *n* = 144), all of which were significant: SM, *F*(3, 481) = 6.750, *p* < 0.001, ηp^2^ = 0.040; EI, *F*(3, 481) = 6.269, *p* < 0.001, ηp^2^ = 0.038; SC, *F*(3, 481) = 4.821, *p* = 0.003, ηp^2^ = 0.029; AB, *F*(3, 481) = 6.647, *p* < 0.001, ηp^2^ = 0.040. Group means, respectively, were SM (3.27, 3.30, 3.49, 3.71), EI (3.56, 3.68, 3.77, 3.99), SC (3.54, 3.65, 3.79, 3.87), and AB (2.35, 2.15, 2.18, 1.93). Tukey *post-hoc* tests showed that for SM and EI the 21–25 group scored higher than the 18–19 groups, with the 20-year group typically intermediate; For SC, the 21–25 group scored significantly higher than the 18-year group; the 20-year group was descriptively higher than the younger groups, but these differences did not reach statistical significance; For AB, the 21–25 group showed the lowest mean aggression; Tukey tests indicated that this group scored significantly lower than the 18-year group, whereas differences relative to the 19- and 20-year groups were in the same direction but did not reach statistical significance. Taken together, older students reported higher SM, EI, and SC and lower AB, which may reflect developmental and contextual differences across undergraduate years. Given these age-related differences, age was included as a covariate in the mediation analyses, and future work could test whether academic year or developmental factors moderate the proposed pathways.

**Table 3:** Age differences in sport motivation, emotional intelligence, self-control, and aggressive behavior (*N* = 485).

Variable	Group 1 (18) M ± SD (*n* = 121)	Group 2 (19) (*n* = 109)	Group 3 (20) (*n* = 111)	Group 4 (21–25) (*n* = 144)	*F*(3, 481)	Partial η^2^
SM	3.27 ± 0.92	3.30 ± 0.83	3.49 ± 0.99	3.71 ± 0.83	6.750	0.040
EI	3.56 ± 0.81	3.68 ± 0.82	3.77 ± 0.90	3.99 ± 0.81	6.269	0.038
SC	3.54 ± 0.82	3.65 ± 0.71	3.79 ± 0.80	3.87 ± 0.71	4.821	0.029
AB	2.35 ± 0.75	2.15 ± 0.85	2.18 ± 0.80	1.93 ± 0.77	6.647	0.040

One-way ANOVAs; degrees of freedom for all *F*-tests are (3, 481). Group codes: 1 = 18 years; 2 = 19 years; 3 = 20 years; 4 = 21–25 years. Total *N* = 485. *p*-values: SM < 0.001; EI < 0.001; SC = 0.003; AB < 0.001.

### 3.4 Serial mediation via emotional intelligence and self-control

**Figure 2:**
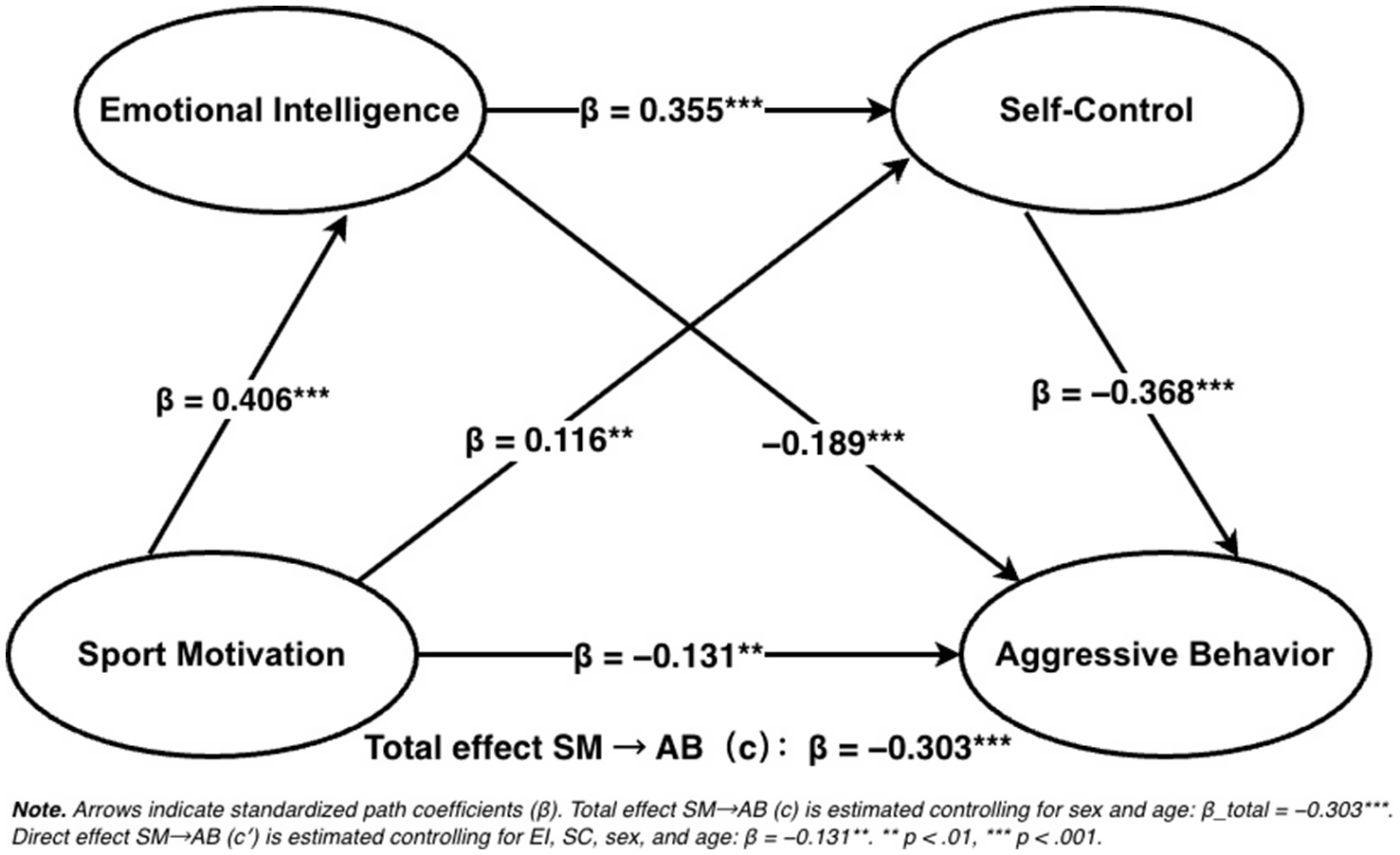
Standardized path coefficients for the serial mediation model (PROCESS Model 6) linking sport motivation with aggressive behavior via emotional intelligence and self-control (EI → SC), controlling for sex and age. SM, sport motivation; EI, emotional intelligence; SC, self-control; AB, aggressive behavior. **p < 0.01, ***p < 0.001.

Confirmatory factor analyses in AMOS 26 for the four core constructs indicated acceptable measurement fit, providing a basis for subsequent path testing (χ^2^/df = 1.368, RMSEA = 0.028, CFI = 0.972, TLI = 0.971). We then tested the serial mediation using PROCESS Model 6 with 5,000 percentile-bootstrap resamples while controlling for sex and age; the regression equations are summarized in Table 4 (standardized coefficients shown in Figure 2), and the total/direct/indirect effects are reported in Table 5. Without mediators, Sport Motivation (SM) was negatively associated with Aggressive Behavior (AB), total effect *B* = −0.267, 95% CI [−0.342, −0.193] (*β* = −0.303). With Emotional Intelligence (EI) and Self-Control (SC) entered simultaneously, the direct effect remained significant and negative, *B* = −0.115, 95% CI [−0.188, −0.042] (*β* = −0.131), indicating partial mediation. In the regression equations (Table 4 and Figure 2), SM was positively associated with EI (*B* = 0.378, SE = 0.038, *β* = 0.406, *t* = 9.984, *p* < 0.001) and with SC (*B* = 0.098, SE = 0.038, *β* = 0.116, *t* = 2.614, *p* = 0.009), and EI was positively associated with SC (*B* = 0.323, SE = 0.041, *β* = 0.355, *t* = 7.836, *p* < 0.001). With AB as the outcome (Table 4 and Figure 2), the negative association for SC was largest in magnitude (*B* = −0.383, SE = 0.045, *β* = −0.368, *t* = −8.536, *p* < 0.001), followed by EI (*B* = −0.179, SE = 0.043, *β* = −0.189, *t* = −4.163, *p* < 0.001), and SM remained negatively associated with AB (*B* = −0.115, SE = 0.037, *β* = −0.131, *t* = −3.098, *p* = 0.002). All three indirect effects were significant (Table 5): via EI, B = −0.068, 95% CI [−0.109, −0.030]; via SC, *B* = −0.038, 95% CI [−0.070, −0.008]; and through the serial EI → SC path, *B* = −0.047, 95% CI [−0.071, −0.028]. Combined, indirect effects summed to *B* = −0.152, 95% CI [−0.199, −0.106], accounting for approximately 56.87% of the total association. Overall, EI and SC each accounted for significant indirect associations between SM and AB, and the serial indirect effect in the specified order (EI → SC) was also significant; thus, the results were consistent with the independent and serial mediation hypotheses (H2, H3, and H4) in this sample.

**Table 4:** Regression analysis of the serial mediation model.

Variable	Emotional intelligence		Self-control		Aggressive behavior		Overall effect	
	*β*	*t*	*β*	*t*	*β*	*t*	*β*	*t*
Sport motivation	0.406	9.984***	0.116	2.614**	−0.131	−3.098**	−0.303	−7.072***
Emotional intelligence	—	—	0.355	7.836***	−0.189	−4.163***	—	—
Self-control	—	—	—	—	−0.368	−8.536***	—	—
*R* ^2^	0.247		0.259		0.340		0.162	
*F*	52.496***		41.946***		49.316***		31.095***	

Standardized coefficients (β) reported. Sex and Age were included as covariates in all models (not shown). **p < 0.01, ***p < 0.001.

**Table 5:** Indirect and direct effects of sport motivation on aggressive behavior via emotional intelligence and self-control.

Path	Unstandardized effect (B)	Proportion of total effect	95% CI	
			LL	UL
SM → EI → AB	−0.068	25.32%	−0.109	−0.030
SM → SC → AB	−0.038	14.07%	−0.070	−0.008
SM → EI → SC → AB	−0.047	17.48%	−0.071	−0.028
Total indirect	−0.152	56.87%	−0.199	−0.106
Direct effect (c′)	−0.115	—	−0.188	−0.042
Total effect (c)	−0.267	—	−0.342	−0.193

Effects are unstandardized (B) and reported with percentile bootstrap 95% confidence intervals based on 5,000 resamples. Sex and age were included as covariates in all equations. Proportions are relative to the total effect c = −0.267 (computed as indirect/c). For reference, completely standardized indirect effects (β_cs) were: total = −0.173 [−0.225, −0.121]; via EI = −0.077 [−0.122, −0.034]; via SC = −0.043 [−0.080, −0.009]; via EI → SC = −0.053 [−0.080, −0.032].

### 3.5 Sensitivity analyses across alternative operationalizations

Sensitivity analyses are summarized in Supplementary Tables S2–S4. The serial indirect effect remained statistically significant across alternative operationalizations of sport motivation, including each SMS-II regulation and the autonomous/controlled motivation indices (serial indirect effects ranged from *B* = −0.042 to −0.047, bootstrap 95% CIs excluding zero). When amotivation was examined in its raw direction, the total and indirect effects reversed direction (positive), consistent with its conceptual meaning as reduced motivation. Replacing the overall EI composite with each WLEIS branch (SEA, OEA, UOE, ROE) yielded comparable and significant serial indirect effects (B range = −0.037 to −0.043). Likewise, replacing the overall self-control composite with each SCS-C19 facet (IC, HH, RT, WF, AE) yielded significant serial indirect effects (B range = −0.034 to −0.042). Collectively, these checks indicate that the main inference is not dependent on a single operationalization of sport motivation, emotional intelligence, or self-control.

## 4 Discussion

In line with our hypotheses, sport motivation was associated with lower aggressive behavior. This association was partly accounted for by emotional intelligence and self-control in the theorized serial sequence from EI to SC. This pattern is consistent with a motivation–emotion–control framework in which emotion-related skills may facilitate inhibitory capacity, thereby reducing aggression-relevant tendencies. Given the cross-sectional, self-report design, these findings should be interpreted as model-based associations rather than evidence of temporal precedence or causality. The following subsections interpret the SM–AB link and the indirect pathways. They also highlight boundary conditions and priorities for longitudinal and intervention research.

### 4.1 Sport motivation and aggressive behavior

Consistent with H1, sport motivation was inversely associated with aggressive behavior in this undergraduate sample. This pattern aligns with evidence that structured sport and activity programs are, on average, linked to lower aggression, while effects remain contingent on context and implementation (Yang et al., 2023; Castillo-Eito et al., 2020; Ouyang and Liu, 2023). From an SDT perspective, stronger internalization and value congruence can promote rule adherence and prosocial orientations. Within the General Aggression Model, normative cues and regulated competition may reduce hostile appraisals and facilitate inhibitory responding. In sport settings, autonomy-supportive or empowering climates have similarly been associated with lower aggressive or antisocial conduct, which is compatible with a motivation-based explanation (Krommidas et al., 2022). Longer-term curricula—including team-based and martial-arts–based programs—also report decreases in aggression-related outcomes, suggesting that repeated exposure to clear rules and self-regulatory demands may be one pathway through which motivation relates to lower aggressive tendencies (Trajković et al., 2020; Shachar et al., 2016; Harwood-Gross et al., 2017).

At the same time, the motivation–aggression association likely depends on how sport is experienced (e.g., activity type, level of contact, supervision, and norm climate). Field and laboratory work indicates that non-contact or rhythmic tasks may dampen anger responses relative to more confrontational formats (Pels and Kleinert, 2016). Reviews also emphasize that well-structured supervision and explicit rule reinforcement are key ingredients associated with fewer conflicts and lower aggression (Spruit et al., 2016). Related comparisons highlight discipline and routinized norms as potentially protective features of organized activity contexts (Pačesová and Šmela, 2020; Basiaga-Pasternak et al., 2020). Because sport type, competitive level, and participation dose were not assessed here, future studies should test these contextual factors as moderators to clarify when higher motivation most strongly relates to lower aggression and when this link may weaken. In campus practice, universities may prioritize supervised, rules-based sport opportunities that emphasize autonomy support and cooperative norms, while applying additional oversight to high-contact formats to avoid normalizing aggressive conduct.

### 4.2 Independent mediation by emotional intelligence and self-control

Consistent with H2, emotional intelligence accounted for part of the association between sport motivation and aggression. One interpretation is that more self-endorsed engagement in sport is accompanied by more frequent practice of emotion appraisal, interpersonal feedback processing, and regulation strategies in structured contexts, which may be reflected in higher EI. This aligns with evidence that sport/physical-activity engagement and motivation are positively related to EI, and that EI-oriented programs in school or PE settings can reduce aggressive or violent behaviors (Ubago-Jiménez et al., 2021; Ortiz-Franco et al., 2024). Intervention and longitudinal work further suggests that strengthening emotion skills—particularly emotional clarity and regulation—can translate into lower subsequent aggression, although effects depend on context and delivery (Garaigordobil and Peña-Sarrionandia, 2015; Castillo et al., 2013; García-Sancho et al., 2017). Mechanistically, the process model of emotion regulation implies that better emotion identification and earlier, more adaptive regulation (e.g., reappraisal) can reduce anger escalation and hostile responding, thereby lowering aggression propensity (Gross, 2015; Bibi et al., 2020; Newman et al., 2021).

Consistent with H3, self-control also functioned as an independent pathway linking sport motivation to lower aggression. Self-control theories posit that inhibitory capacity constrains impulsive reactions under provocation; when self-control resources are higher, aggressive impulses are more likely to be inhibited (Baumeister et al., 2007; DeWall et al., 2011; Sofia and Cruz, 2015; Chan and Chui, 2017). Meta-analytic evidence reports a robust negative association between self-control and aggression, including in university samples, supporting self-control as a proximal resource relevant to aggressive tendencies (Lei et al., 2020). At an applied level, reviews of sport- and activity-based programs indicate that interventions embedding explicit self-control practice and rule reinforcement are often associated with reductions in aggression, although effects vary by design and population (Ouyang and Liu, 2023; Yang et al., 2023; Denson et al., 2012; Trajković et al., 2020). Taken together, these two indirect pathways suggest that emotion-related skills and inhibitory control each plausibly explain part of the motivation–aggression association, while leaving room for additional mechanisms not included in the current model. For practice, physical education courses and student support services may embed brief emotion-skill training and self-control routines into regular sport sessions. Instructors can reinforce emotion labeling, cognitive reappraisal, and impulse inhibition through structured reflection and feedback during common peer-conflict scenarios.

### 4.3 Serial mediation of emotional intelligence and self-control

Consistent with H4, the serial pathway suggests that sport motivation may relate to lower aggression through a coupled sequence of emotional and inhibitory regulation (EI → SC). In structured sport contexts, motivated engagement may provide repeated opportunities for emotion appraisal, social feedback, and regulation practice, which can be reflected in higher emotional intelligence and improved coping-relevant skills. This interpretation is consistent with prior work linking sport engagement or sport-related motivation to higher EI and related adaptive outcomes across samples and settings (Laborde et al., 2016; Ubago-Jiménez et al., 2019; Šukys et al., 2019; Vega et al., 2022). In addition, EI has been associated with self-control and broader self-regulatory functioning in chained associations, supporting the idea that emotion-processing skills may reduce the self-regulatory burden during emotionally charged moments (Gutiérrez-Cobo et al., 2018; Drndarević et al., 2021).

Downstream, self-control represents a more proximal constraint on aggressive impulses, and evidence indicates that higher self-control is linked to lower aggression and related maladaptive behaviors across populations (Li et al., 2017; Yue et al., 2024; Zvi and Lavi, 2025). Because the present data are cross-sectional and self-reported, the EI → SC ordering should be interpreted as a theory-informed specification rather than proof of temporal precedence; reciprocal or reverse processes remain plausible. Future longitudinal and intervention studies can directly test temporal ordering (e.g., whether changes in EI precede changes in self-control) and examine whether the strength of the serial pathway varies across broader student adjustment contexts and stress exposures (La Rosa et al., 2025). In applied settings, universities may pilot sport-based programs that intentionally sequence emotional skill development before repeated self-control practice. Semester-length pilots can monitor feasibility and simple behavioral indicators of conflict and rule violations to inform larger trials.

### 4.4 Limitations and future directions

This study relied on cross-sectional, single-source self-report data and regression-based mediation models; accordingly, the findings should be interpreted as associational and model-based evidence rather than causal effects, even though sex and age were included as covariates in the mediation analyses. To reduce respondent burden and harmonize response formats, all instruments used five-point response options; although internal consistency and CFA fit were acceptable, the response-range modification may affect score variance and comparability with versions using the original response formats. Because all measures were collected at a single time point from the same respondents, common-method variance remains a possibility; Harman’s single-factor test and the one-factor CFA comparison did not indicate a dominant common factor, but residual method bias cannot be ruled out. The sample comprised non–sport-major undergraduates from a single university, limiting generalizability across institutions, regions, and sport backgrounds. Mediation estimates were based on observed composite scores without explicit correction for measurement error and without modeling full multidimensional structures as latent variables. Importantly, sensitivity analyses indicated that the key serial indirect effect was stable across theoretically informed alternative operationalizations of sport motivation (SMS-II regulations and autonomous/controlled indices), EI branches, and self-control facets (Supplementary Tables S2–S4), although these checks do not substitute for latent-variable modeling that explicitly accounts for measurement error. Finally, we did not measure sport participation dose, sport type, competitive level, or contextual features such as supervision and motivational climate, which limits interpretation of boundary conditions.

Future research should strengthen causal inference and clarify directionality using multi-wave longitudinal designs, cross-lagged approaches, and intervention studies that can test whether changes in emotional intelligence precede changes in self-control and subsequent aggression. Measurement can be improved by incorporating multi-informant reports, behavioral tasks indexing emotion processing and inhibitory control, and objective indicators of sport participation alongside records of conflicts or rule violations. Analytically, latent-variable SEM can test whether specific motivational regulations, EI branches, or self-control facets drive the indirect pathways while explicitly accounting for measurement error, and competing specifications such as SC preceding EI can be compared directly. Studies should also examine moderation by sport type, contact level, supervision quality, and competitive context to identify when sport motivation is most strongly linked to lower aggression. Translationally, universities can pilot autonomy-supportive sport environments that embed brief emotion-skill and self-control routines, and evaluate feasibility and maintenance across student subgroups.

## 5 Conclusion and recommendations

Drawing on a sample of Chinese undergraduates from non–sport majors, this study found a significant negative association between sport motivation and aggressive behavior. In regression-based serial mediation models controlling for sex and age, emotional intelligence and self-control each accounted for significant indirect associations, and the serial indirect association in the specified order (EI → SC) was also significant. The combined indirect associations accounted for 56.87% of the total association, with the serial pathway contributing approximately 18%; this inference was robust across alternative operationalizations of sport motivation based on SMS-II subscales and autonomous/controlled composites, whereas the direction reversed for amotivation when scored in its raw direction. Overall, the pattern is consistent with self-determination, emotion-regulation, and self-control perspectives within the broader aggression framework, suggesting that sport motivation is associated with lower aggression partly via emotion-related skills and inhibitory control resources. Given the cross-sectional self-report design, these findings should be interpreted as associational and model-based, and longitudinal or experimental studies are needed to test temporal ordering and causal mechanisms.

Building on these findings, universities may consider leveraging physical education and student support systems to foster autonomy-supportive sport contexts while incorporating brief emotion- and self-control–focused skill practices. Programs could prioritize non-contact, cooperative activities and provide clear rules and timely feedback; for higher-contact activities, additional components such as rule rehearsal, officiating literacy, and de-escalation drills may be warranted. Simple in-class micro-practices (e.g., emotion labeling and reappraisal; if–then plans, self-monitoring, and delay-of-gratification exercises) could be embedded and evaluated using brief scales and incident logs across the semester. Such approaches should be tested for feasibility, scalability, and subgroup differences (sex, academic year, sport experience) before being recommended as evidence-based interventions.

## Data Availability

The original contributions presented in the study are included in the article/Supplementary material, further inquiries can be directed to the corresponding author/s.
