# Supplementary material for: Sport motivation is associated with lower aggression via emotional intelligence and self-control: a serial mediation study in undergraduates
Source: Front Psychol. 2026 Feb 26;17:1762835. doi: 10.3389/fpsyg.2026.1762835 (PMC13015190; doi:10.3389/fpsyg.2026.1762835)
Supplement: Supplementary file 2 [file Table_2.DOCX]

**Supplementary Material S1. Survey Questionnaire (Chinese–English)**

**Note:** This file reproduces the exact questionnaire items used in the study and presents each item in English followed by Chinese. All scale items were answered on a 5-point Likert scale (1 = Strongly disagree, 5 = Strongly agree) unless otherwise specified. Items marked “(R)” were reverse-scored for analysis.

# A. Participant Information and Consent / 研究说明与知情同意

Dear student,
Thank you for participating in this survey on the relationship between sport motivation and aggressive behavior among undergraduates. Your views are valuable for improving campus sport development and sustainable management. Please answer based on your true feelings.

Participation is completely voluntary. You may withdraw at any time. Responses that are not submitted will not be saved.

This questionnaire does not collect any personally identifiable information (e.g., name or contact details). All data are used only for academic research and campus policy improvement and will not be disclosed to any third party.

The survey takes approximately 5–10 minutes. All questions are multiple-choice or rating-scale items; there are no right or wrong answers.

The study protocol was reviewed and approved by the Ethics Committee (IRB No.: KJ2025-520-01) and was conducted in accordance with the Declaration of Helsinki.

By clicking “Start” or “Next”, you indicate that you have read and agreed to the above information. Thank you for your time.

亲爱的同学，您好！
非常感谢您愿意参与本次关于体育动机对大学生攻击行为的调查。您的观点对学校体育运动发展和可持续管理具有重要价值。请在阅读以下说明后，依据个人真实感受填写问卷。

本调查完全基于自愿原则，您可随时退出，未提交的回答将不会被保存。

问卷不收集姓名、联系方式等任何可识别个人身份的信息，所有数据仅用于学术研究及校园政策改进，绝不向第三方泄露。

填答时长约为 5–10 分钟，问题均为选择或量表形式，没有对错之分。

研究已通过伦理委员会审查（IRB 编号：KJ2025-520-01），并遵循《赫尔辛基宣言》相关原则。

点击“开始”或“下一页”即表示您已阅读并同意以上说明。再次感谢您的宝贵时间，祝您生活愉快！

# B. Demographics / 基本信息

1. Gender: Male / Female

1. 性别: 男 / 女

2. Age: 18; 19; 20; 21 or above

2. 年龄: 18岁；19岁；20岁；21岁及以上

Response scale for the following items: 1 = Strongly disagree, 2 = Disagree, 3 = Neither agree nor disagree, 4 = Agree, 5 = Strongly agree.

以下量表题目均采用 5 点计分：1 = 非常不同意，2 = 不同意，3 = 一般，4 = 同意，5 = 非常同意。

# C. Sport Motivation Scale II (SMS-II; 18 items) / 体育动机量表（SMS-II；18题）

1. Because I would feel bad about myself if I did not take the time to do it.

因为不参加这项运动，我会觉得自己不好。

2. I used to have good reasons for doing sports, but now I am asking myself if I should continue.

我曾经有很充分的理由参加这项运动，但现在开始怀疑自己是否还要继续。

3. Because it is very interesting to learn how I can improve.

因为学习如何提高自己在这项运动中的表现非常有趣。

4. Because practicing sports reflects the essence of whom I am.

因为参与这项运动体现了真正的自我。

5. Because people I care about would be upset with me if I didn’t.

因为如果我不参加这项运动，身边重要的人会对我不高兴。

6. Because I found it is a good way to develop aspects of myself that I value.

因为参加这项运动是提升我所看重的自身素质的一个好方式。

7. Because I would not feel worthwhile if I did not.

因为如果我不参加这项运动，我会觉得自己没有价值。

8. Because I think others would disapprove of me if I did not.

因为如果我不参加这项运动，别人会对我表示不满。

9. Because I find it enjoyable to discover new performance strategies.

因为发现提高这项运动表现的新办法让我感到愉快。

10. I don’t know anymore; I have the impression that I am incapable of succeeding in this sport.

我已经不知道为什么要参加这项运动了，我觉得自己在这项运动中很难取得成功。

11. Because participating in sport is an integral part of my life.

因为参加这项运动已经成为我生活中不可或缺的一部分。

12. Because I have chosen this sport as a way to develop myself.

因为我选择这项运动作为自我发展的途径。

13. It is not clear to me anymore; I don’t really think my place is in this sport.

现在我已经说不清为什么要参加这项运动，我并不真正认为自己适合这项运动。

14. Because through sport, I am living in line with my deepest principles.

因为通过这项运动，我能够按照自己最深层的原则来生活。

15. Because people around me reward me when I do.

因为当我参加这项运动时，周围的人会给予我奖励。

16. Because I feel better about myself when I do.

因为当我参加这项运动时，我会对自己感觉更好。

17. Because it gives me pleasure to learn more about my sport.

因为进一步了解这项运动会让我感到愉快。

18. Because it is one of the best ways I have chosen to develop other aspects of myself.

因为参加这项运动是我用来发展自己其他方面能力的最佳方式之一。

# D. Wong and Law Emotional Intelligence Scale (WLEIS; 16 items) / 情绪智力量表（WLEIS；16题）

1. I have a good sense of why I have certain feelings most of the time.

我在大多数时候都很清楚自己为什么会产生某些情绪。

2. I have a good understanding of my own emotions.

我对自己的情绪有很好的理解。

3. I really understand what I feel.

我确实明白自己的感受。

4. I always know whether or not I am happy.

我总是知道自己是否感到快乐。

5. I always know my friends’ emotions from their behaviour.

我总能从朋友的行为中看出他们的情绪。

6. I am a good observer of others’ emotions.

我善于观察别人的情绪。

7. I am sensitive to the feelings and emotions of others.

我对他人的感受和情绪很敏感。

8. I have a good understanding of the emotions of people around me.

我对周围人的情绪理解得很好。

9. I always set goals for myself and then try my best to achieve them.

我总是为自己设定目标，并尽最大努力去实现。

10. I always tell myself I am a competent person.

我总会告诉自己我是一个有能力的人。

11. I am a self-motivated person.

我是一个会自我激励的人。

12. I would always encourage myself to try my best.

我总会鼓励自己尽最大努力。

13. I am able to control my temper and handle difficulties rationally.

我能够控制自己的脾气，并理性地处理困难。

14. I am quite capable of controlling my own emotions.

我相当能够控制自己的情绪。

15. I can always calm down quickly when I am very angry.

当我非常生气时，我总能很快让自己平静下来。

16. I have good control of my own emotions.

我很好地掌控着自己的情绪。

# E. Self-Control Scale (SCS-C19; 19 items) / 自制力量表（SCS-C19；19题）

1. I can resist temptation very well.

我能很好地抵制诱惑。

2. It is difficult for me to break bad habits.

对我来说改掉坏习惯是困难的。

3. I am lazy.

我是懒惰的。

4. I sometimes do things that bring me pleasure but are harmful to myself.

我会做一些能给自己带来快乐但对自己有害的事情。

5. People believe I can stick to an action plan.

人们相信我能坚持行动计划。

6. Getting up in the morning is difficult for me.

对我来说，早上起床是件困难的事。

7. People say I am impulsive.

大家说我是冲动的。

8. I spend money too freely.

我太能花钱了。

9. I become so emotional that I lose control.

我会因为情感而激动得不能自持。

10. Many of the things I do are done on impulse.

我做的很多事情是因为一时冲动。

11. People say I have iron self-control.

大家说我有钢铁般的自制力。

1. Sometimes I am distracted by enjoyable things and fail to finish tasks on time.

有时我会被有乐趣的事情干扰而不能按时完成任务。

13. I find it hard to concentrate.

我难以集中注意力。

14. I can work efficiently toward a long-term goal.

我能为了一个长远目标高效地工作。

15. Sometimes I cannot help doing things even when I know they are wrong.

有时我会忍不住去做一些事情，即使我知道那样做是错误的。

16. I often act without thinking things through.

我常常考虑不周就付诸行动。

17. I lose my temper too easily.

我太容易发脾气。

18. I often interrupt others.

我经常打扰别人。

19. I sometimes drink alcohol (or go online) excessively.

我有时会饮酒（或上网）过度。

# F. Brief Aggression Questionnaire (BAQ-12; 12 items) / 简式攻击行为问卷（BAQ-12；12题）

1. Given enough provocation, I may hit another person.

若受到足够的挑衅，我可能会打人。

2. If I have to resort to violence to protect my rights, I will.

如果必须诉诸暴力来维护自己的权利，我会这么做。

3. There are people who pushed me so far that we came to blows.

有些人曾把我逼得太狠，以至于我们最终动起了手。

4. I am an even-tempered person.

我是一个性情平和的人。

5. Sometimes I fly off the handle for no good reason.

有时我会无缘无故地大发脾气。

6. I have trouble controlling my temper.

我很难控制自己的脾气。

7. I tell my friends openly when I disagree with them.

当我与朋友意见不一致时，我会坦率地说出来。

8. When people annoy me, I may tell them what I think of them.

当有人惹恼我时，我可能会直接告诉他们我对他们的看法。

9. My friends say that I’m somewhat argumentative.

我的朋友说我有点爱争论。

10. Other people always seem to get the breaks.

别人似乎总是比我更走运 / 更容易得到好机会。

11. I sometimes feel that people are laughing at me behind my back.

有时我觉得别人会在背后笑话我。

12. When people are especially nice, I wonder what they want.

当别人对我格外好时，我会怀疑他们想从我这里得到什么。
