# Supplementary material for: Sport motivation is associated with lower aggression via emotional intelligence and self-control: a serial mediation study in undergraduates
Source: Front Psychol. 2026 Feb 26;17:1762835. doi: 10.3389/fpsyg.2026.1762835 (PMC13015190; doi:10.3389/fpsyg.2026.1762835)
Supplement: Supplementary file 3 [file Table_3.DOCX]

Supplementary Material

# Supplementary Tables

**Supplementary Table S1.** Confirmatory factor analysis (CFA) model comparison used to evaluate potential common-method variance (CMV).

Comparison of a one-factor CFA model (all 19 observed indicators loading on a single latent factor) with the hypothesized four-factor measurement model (SM, EI, SC, and AB).

| **Model** | **χ²**  **(CMIN)** | **df** | **χ²/df** | **CFI** | **TLI** | **IFI** | **NFI** | **RMSEA [90% CI]** | **RMR** | **AIC** |
| --- | --- | --- | --- | --- | --- | --- | --- | --- | --- | --- |
| One-factor | 6618.395 | 152 | 43.542 | .444 | .375 | .445 | .440 | .296 [.290, .303] | .197 | 6694.395 |
| Multi-factor (hypothesized) | 168.456 | 146 | 1.154 | .998 | .998 | .998 | .986 | .018 [.000, .029] | .012 | 256.456 |

***Note.*** χ² = CMIN from AMOS. Indicators were subscale mean scores: SMS-II (6 regulations), WLEIS (4 EI branches), SCS-C19 (5 self-control facets), and BAQ (4 aggression facets). RMSEA 90% confidence intervals are the LO 90 and HI 90 bounds reported by AMOS. RMR = root mean square residual. AIC = Akaike information criterion. Estimation method: maximum likelihood. N = 485.

**Supplementary Table S2.** Sensitivity analysis across alternative SMS-II operationalizations of sport motivation (X), with EI→SC serial mediation.

| **Predictor**  **(X)** | **Total effect c, B [95% CI]** | **Direct effect c', B [95% CI]** | **Total indirect**  **(c−c'), B** | **Serial indirect**  **(X→EI→SC→AB), B [Boot 95% CI]** | **Serial / total**  **(%)** |
| --- | --- | --- | --- | --- | --- |
| IM | -0.249 [-0.320, -0.177] | -0.108 [-0.178, -0.038] | -0.141 | -0.045 [-0.069, -0.027] | 18.07 |
| INTEG | -0.238 [-0.309, -0.168] | -0.106 [-0.174, -0.038] | -0.132 | -0.042 [-0.064, -0.025] | 17.65 |
| IDEN | -0.221 [-0.292, -0.150] | -0.079 [-0.148, -0.010] | -0.142 | -0.044 [-0.067, -0.026] | 19.91 |
| INTROJ | -0.251 [-0.321, -0.181] | -0.112 [-0.180, -0.043] | -0.139 | -0.042 [-0.063, -0.024] | 16.73 |
| EXTERN | -0.242 [-0.312, -0.172] | -0.106 [-0.174, -0.039] | -0.136 | -0.042 [-0.063, -0.025] | 17.36 |
| AM (raw) | 0.229 [0.158, 0.299] | 0.091 [0.023, 0.159] | 0.138 | 0.041 [0.024, 0.062] | 17.90 |
| Aut (composite) | -0.260 [-0.334, -0.186] | -0.109 [-0.182, -0.037] | -0.151 | -0.047 [-0.071, -0.028] | 18.08 |
| Con (composite) | -0.264 [-0.336, -0.192] | -0.118 [-0.189, -0.048] | -0.146 | -0.044 [-0.067, -0.026] | 16.67 |

***Note.*** Each row represents a separate PROCESS Model 6 analysis with aggressive behavior (AB) as Y, emotional intelligence (EI; overall composite) as M1, self-control (SC; overall composite) as M2, and sex and age as covariates. Coefficients are unstandardized (B). Total effect (c) and direct effect (c′) are reported with 95% confidence intervals. Indirect effects are reported with percentile bootstrap 95% confidence intervals based on 5,000 resamples. Total indirect = c − c′. Serial/total (%) = (serial indirect / total effect) × 100. SMS-II subscales: IM = intrinsic motivation; INTEG = integrated regulation; IDEN = identified regulation; INTROJ = introjected regulation; EXTERN = external regulation; AM (raw) = amotivation scored in its original direction (not reverse-coded). Autonomous composite = mean of IM, INTEG, and IDEN; Controlled composite = mean of INTROJ and EXTERN. N = 485.

**Supplementary Table S3.** Sensitivity analysis across EI branches as the first mediator (M1), with overall self-control as the second mediator (M2).

| **M1 (EI branch)** | **M2** | **Total effect c, B [95% CI]** | **Direct effect c', B [95% CI]** | **Indirect via M1, B [Boot 95% CI]** | **Indirect via M2, B [Boot 95% CI]** | **Serial indirect, B [Boot 95% CI]** | **Total indirect, B [Boot 95% CI]** | **Serial / total (%)** |
| --- | --- | --- | --- | --- | --- | --- | --- | --- |
| SEA | SC | -0.267 [-0.342, -0.193] | -0.130 [-0.202, -0.057] | -0.051 [-0.086, -0.017] | -0.045 [-0.079, -0.015] | -0.042 [-0.064, -0.025] | -0.138 [-0.186, -0.093] | 15.82 |
| OEA | SC | -0.267 [-0.342, -0.193] | -0.125 [-0.198, -0.052] | -0.056 [-0.093, -0.020] | -0.044 [-0.079, -0.014] | -0.043 [-0.065, -0.025] | -0.142 [-0.192, -0.097] | 16.05 |
| UOE | SC | -0.267 [-0.342, -0.193] | -0.129 [-0.201, -0.057] | -0.051 [-0.085, -0.020] | -0.049 [-0.083, -0.020] | -0.038 [-0.058, -0.022] | -0.138 [-0.184, -0.095] | 14.19 |
| ROE | SC | -0.267 [-0.342, -0.193] | -0.117 [-0.190, -0.044] | -0.063 [-0.102, -0.029] | -0.051 [-0.088, -0.020] | -0.037 [-0.058, -0.020] | -0.150 [-0.197, -0.108] | 13.66 |

Note. Each row represents a separate PROCESS Model 6 analysis with sport motivation (SM; overall 18-item index (amotivation reverse-coded)) as X and aggressive behavior (AB) as Y. M1 is the specified WLEIS branch and M2 is the overall self-control composite. Sex and age were included as covariates. Coefficients are unstandardized (B). Total effect (c) and direct effect (c′) are reported with 95% confidence intervals. Indirect effects are reported with percentile bootstrap 95% confidence intervals based on 5,000 resamples. Total indirect = c − c′. Serial/total (%) = (serial indirect / total effect) × 100. WLEIS branches: SEA = self-emotion appraisal; OEA = others’ emotion appraisal; UOE = use of emotion; ROE = regulation of emotion. N = 485.

**Supplementary Table S4.** Sensitivity analysis across self-control facets as the second mediator (M2), with overall EI as the first mediator (M1).

| **M1** | **M2 (SC facet)** | **Total effect c, B [95% CI]** | **Direct effect c', B [95% CI]** | **Indirect via M1, B [Boot 95% CI]** | **Indirect via M2, B [Boot 95% CI]** | **Serial indirect, B [Boot 95% CI]** | **Total indirect, B [Boot 95% CI]** | **Serial / total (%)** |
| --- | --- | --- | --- | --- | --- | --- | --- | --- |
| EI | IC | -0.267 [-0.342, -0.193] | -0.119 [-0.192, -0.045] | -0.072 [-0.113, -0.033] | -0.034 [-0.066, -0.007] | -0.042 [-0.065, -0.024] | -0.149 [-0.198, -0.105] | 15.70 |
| EI | HH | -0.267 [-0.342, -0.193] | -0.126 [-0.200, -0.052] | -0.079 [-0.118, -0.041] | -0.027 [-0.056, -0.002] | -0.036 [-0.054, -0.020] | -0.141 [-0.188, -0.098] | 13.36 |
| EI | RT | -0.267 [-0.342, -0.193] | -0.121 [-0.195, -0.047] | -0.076 [-0.115, -0.041] | -0.032 [-0.063, -0.006] | -0.038 [-0.058, -0.022] | -0.147 [-0.192, -0.105] | 14.28 |
| EI | WF | -0.267 [-0.342, -0.193] | -0.120 [-0.193, -0.047] | -0.072 [-0.111, -0.034] | -0.033 [-0.062, -0.006] | -0.042 [-0.064, -0.025] | -0.147 [-0.194, -0.102] | 15.76 |
| EI | AE | -0.267 [-0.342, -0.193] | -0.126 [-0.201, -0.050] | -0.080 [-0.121, -0.042] | -0.027 [-0.056, -0.004] | -0.034 [-0.054, -0.018] | -0.142 [-0.188, -0.097] | 12.78 |

**Note.** Each row represents a separate PROCESS Model 6 analysis with sport motivation (SM; overall 18-item index (amotivation reverse-coded)) as X and aggressive behavior (AB) as Y. M1 is the overall emotional intelligence (EI) composite and M2 is the specified self-control facet from the SCS-C19. Sex and age were included as covariates. Coefficients are unstandardized (B). Total effect (c) and direct effect (c′) are reported with 95% confidence intervals. Indirect effects are reported with percentile bootstrap 95% confidence intervals based on 5,000 resamples. Total indirect = c − c′. Serial/total (%) = (serial indirect / total effect) × 100. SCS-C19 facets: IC = impulse control; HH = healthy habits; RT = resisting temptation; WF = focus on work; AE = moderation in recreation. N = 485.
